# Nilotinib in Parkinson's disease: A systematic review and meta-analysis

**DOI:** 10.3389/fnagi.2022.996217

**Published:** 2022-09-29

**Authors:** Xiaolu Xie, Ping Yuan, Liqiu Kou, Xiu Chen, Jun Li, Yaling Li

**Affiliations:** ^1^Department of Pharmacy, The Affiliated Hospital of Southwest Medical University, Luzhou, China; ^2^School of Pharmacy, Southwest Medical University, Luzhou, China; ^3^Department of Neurology, The Affiliated Hospital of Southwest Medical University, Luzhou, China; ^4^Department of Traditional Chinese Medicine, The Affiliated Hospital of Southwest Medical University, Luzhou, China

**Keywords:** nilotinib, Parkinson's disease, tolerability, MDS-UPDRS, safety, CSF biomarker levels

## Abstract

**Background:**

Nilotinib, which inhibits cellular Abelson tyrosine kinase, may be an effective treatment for patients with Parkinson's disease (PD). The purpose of this study is to evaluate the outcomes of different doses of nilotinib in patients with PD.

**Methods:**

We searched PubMed, Embase, Web of Science, and Cochrane Central Register of Controlled Clinical Trials from inception to 7 March 2022 to identify all randomized controlled trials (RCTs) of nilotinib reporting outcomes of interest in patients with PD. Outcomes included tolerability, efficacy, safety, and CSF biomarker levels. Review manager 5.4 software was used to analyze all data.

**Results:**

Three RCTs with a total of 163 patients were included. No significant difference was found between 150 mg nilotinib or 300 mg nilotinib and placebo in terms of tolerability, adverse events, or HVA levels. 300 mg nilotinib showed significantly higher Movement Disorder Society Unified Parkinson's Disease Rating Scale III (MDS-UPDRS III) scores [SMD = 0.52, 95%CI = (0.12, 0.92), *P* = 0.01] and 3,4-dihydroxyphenylacetic acid (DOPAC) levels [SMD = 0.52, 95%CI = (0.12, 0.92), *P* = 0.01], and lower α-synuclein levels [SMD = −2.16, 95%CI = (−3.38, −1.84), *P* < 0.00001] compared with placebo. And compared with 150 mg nilotinib, 300 mg nilotinib showed significantly lower α-synuclein levels [SMD = −1.16, 95%CI = (−1.70, −0.61), *P* < 0.0001].

**Conclusions:**

Although our study demonstrated favorable tolerability and safety of different doses of nilotinib, and improvement in part of CSF biomarker levels of 300 mg nilotinib, the poor efficacy on motor outcomes indicated that nilotinib had no advantages in the clinic.

## Introduction

Parkinson's disease (PD) is the second most common neurodegenerative disease, of which the prevalence and disability have more than doubled over the past two decades, affecting more than 6 million individuals worldwide (Bloem et al., 2021; Tolosa et al., 2021). PD is an age-related progressive disorder, pathologically characterized by the loss of dopaminergic neurons in the pars compacta of the substantia nigra and by the accumulation of α-synuclein in Lewy bodies and Lewy neuritis (Bloem et al., 2021). Parkin also plays a pivotal role in PD pathogenesis, and its inactivation can aggravate the accumulation of α-synuclein and accelerate the progression of PD (Ganguly et al., 2020; Madsen et al., 2021). Primary motor symptoms of PD include tremor, rigidity, bradykinesia, gait and posture alterations. Current therapies are symptomatic and primarily focus on dopamine replacement strategies and effective relief of motor dysfunctions (Balestrino and Schapira, 2020). Although dopamine replacement strategies, including levodopa, dopamine agonists and monoamine oxidase type B (MAO-B) inhibitors, are beneficial in the early stages of disease, they can't slow or stop disease progression (Balestrino and Schapira, 2020; Bloem et al., 2021). Moreover, long-term levodopa treatment relates to the development of motor complications such as fluctuations, dyskinesia and freezing, as well as other non-motor side-effects due to decreased tolerance (Balestrino and Schapira, 2020; Werner and Olanow, 2022). Slowing and stopping PD progression pathologically and reducing relevant clinical manifestations remain a major unmet need in the treatment of PD.

Nilotinib, an oral cellular Abelson tyrosine kinase (c-Abl) inhibitor, was approved for the treatment of chronic myeloid leukemia (CML) at dosages of 300 mg twice daily by the U.S FDA in 2007 (Deremer et al., 2008; Sacha and Saglio, 2019). Some studies found increased activation of c-Abl in PD models and in brain tissues of PD patients (Imam et al., 2011; Brahmachari et al., 2016, 2019; Karim et al., 2020; Ghosh et al., 2021). suggesting that c-Abl might be associated with PD progression and that its inhibitor nillotinib might have a potential benefit in treating PD. Subsequent studies found that nilotinib (1–10 mg/kg) could protect animal models of PD from neurodegeneration in the brain via inhibiting c-Abl, degrading α-synuclein and blocking inactivation of parkin (Hebron et al., 2013a, 2014; Karuppagounder et al., 2014; Lonskaya et al., 2014; Wu et al., 2021; Werner and Olanow, 2022). Researchers also found that nilotinib could improve motor behavior in PD models (Hebron et al., 2013a). These findings sparked interest in whether nilotinib can slow PD progression clinically. In 2016, comparing 150 mg nilotinib with 300 mg nilotinib, a small clinical trial of 12 patients with advanced PD and dementia with Lewy bodies firstly demonstrated that nilotinib could effectively improve cerebrospinal fluid (CSF) biomarker levels and had a beneficial effect on motor and cognition outcomes (Pagan et al., 2016). Subsequently, two clinical trials on tolerance, efficacy, safety and biomarkers of multi-dose nilotinib were conducted (Pagan et al., 2020; Simuni et al., 2021). However, there is still a lack of a systematic review to synthesize existing evidence for a definitive conclusion about whether nilotinib is a clinically effective treatment and the appropriate dose of nilotinib for clinical use.

Therefore, based on the available clinical evidence, we aimed to evaluate differences in tolerability, efficacy, safety and CSF biomarker levels in different doses of nilotinib by this systematic review and meta-analysis, in order to guide the development of more multi-center, large-sample and high-quality clinical trials and broaden its indications.

## Methods

This study followed the Preferred Reporting Items for Systematic reviews and Meta-Analyses (PRISMA) guidelines (Moher et al., 2009).

### Search strategy

We searched PubMed, Embase, Web of science, and the Cochrane Central Register of Controlled Clinical Trials for studies published from database inception to March 7, 2022, using medical subject headings (MeSH) and free words combined with *nilotinib* and *Parkinson*'*s disease*. The full search strategy for Pubmed is included in the Supplementary material.

### Inclusion and exclusion criteria

Studies that fulfilled the following inclusion criteria were included: (1) only randomized controlled trials (RCTs); (2) patients were diagnosed with PD; (3) nilotinib was used in at least 1 treatment arm; (4) the studies reported at least 1 outcome of interest. Studies were excluded as follows: (1) duplicates from the same clinical trial; (2) full text unavailable; (3) unable to extract data.

### Outcomes

There were four outcomes, which included tolerability, efficacy and safety of nilotinib, and CSF biomarker levels in PD patients. Tolerability was defined as the proportion of patients who had the ability to complete the study while receiving the assigned dose. Efficacy was defined as the improvement of motor behavior of patients who had lower Movement Disorder Society Unified Parkinson's Disease Rating Scale III (MDS-UPDRS III) scores and Unified Parkinson's Disease Rating Scale III (UPDRS III) scores. Safety was represented by adverse events (AEs) reported in the included studies, including non-serious adverse events (non-SAEs) and serious adverse events (SAEs). Common non-SAEs included fall, musculoskeletal disorders, skin and subcutaneous disorders, and gastrointestinal disorders. Common SAEs included serious cardiac disorders and serious gastrointestinal disorders. CSF biomarker levels included the concentration of α-synuclein, the dopamine metabolites homovanillic acid (HVA) and 3,4-dihydroxyphenylacetic acid (DOPAC) in the CSF. A decrease in α-synuclein concentration, and an increase in HVA and DOPAC concentrations indicate that nilotinib can improve PD-related pathological features (Hebron et al., 2013a; Pagan et al., 2020).

### Study selection and data extraction

After removing duplications by Endnote X9, two reviewers (XX and KL) independently screened the title and abstract according to the inclusion and exclusion criteria, and then read the full text to determine the final inclusions. For articles from the same clinical trial, we included only the most comprehensive data. We extracted the following data: study characteristics (first author, publication year, study design, intervention and control, and follow-up), baseline demographics of participants (age, sex, diagnostic criteria, Hoehn-Yahr [H/Y] stage, and disease duration), and outcomes of interest. Discrepancies were resolved by a third reviewer (CX).

### Quality assessment

Two reviewers (XX and KL) independently assessed the risk of bias of RCTs with the Cochrane Collaboration's tool, (Higgins et al., 2011) and discrepancies were resolved by a third reviewer (CX).

### Statistical analysis

Review manager software (version 5.4; the Cochrane Collaboration) was used to analysis all data. For dichotomous data (eg, tolerability, AEs), odds ratio (OR) and 95% Confidence interval (CI) were estimated for each study. For continuous data (eg, MDS-UPDRS III, UPDRS III, CSF biomarker levels), standard mean difference (SMD) and 95% CI were calculated as effect indexes. Heterogeneity among individual studies was judged by I^2^ values. We used a fixed effects model when I^2^ <50%; otherwise, we used a random effects model. Publication bias was assessed by an Egger test and a Begg rank correlation (Egger et al., 1997). *P* < 0.05 was considered statistically significant.

## Results

### Study selection

**Figure 1:**
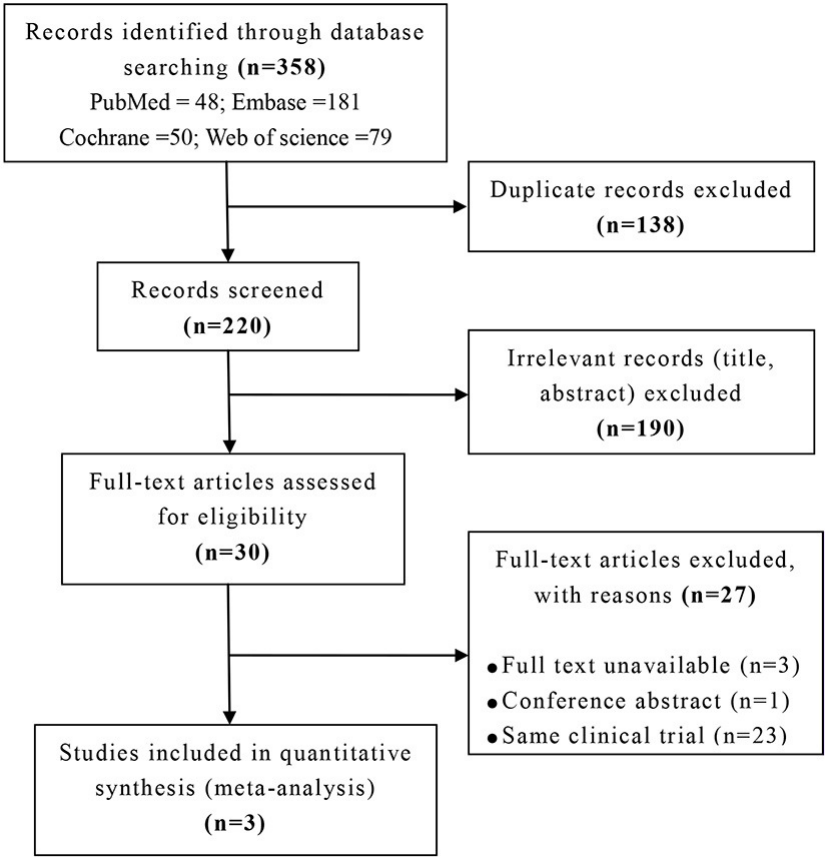
Flow chart of literature searching.

Overall, the retrieval identified 358 studies, 138 duplicates removed, and 220 studies were screened title and abstract. After excluding 190 irrelevant studies, 30 studies were screened full text. Of those, 27 were excluded. Of the excluded studies, 3 were not available, 1 was conference abstract, and 23 were same clinical trials. Ultimately, 3 studies were included for analysis with 163 patients (Pagan et al., 2016, 2020; Simuni et al., 2021). The full selection strategy was presented in Figure 1.

### Study characteristics

All three studies were conducted in the United States. Two studies were single center studies, (Pagan et al., 2016, 2020) and 1 study was multicenter study (Simuni et al., 2021). Two studies were phase 2, double blind, placebo controlled trials, (Pagan et al., 2020; Simuni et al., 2021) and 1 study was phase 1, open label trial without placebo (Pagan et al., 2016). All three studies adopted the UK Brain Bank diagnostic criteria for PD. One study contained patients diagnosed with dementia with lewy bodies (Pagan et al., 2016). All included studies compared 150 mg nilotinib with 300 mg nilotinib. The general characteristics of the included studies were in Table 1.

**Table 1:** General characteristics of the included studies.

**Reference**	**Study design**	**No. of patients**	**Characteristics of study participants**	**Intervention**	**Follow up**	**Outcomes**
Pagan et al. (2016)	Single center, RCT, open label	12	Mean age, y: 150 mg nilotinib (72.4); 300 mg nilotinib (71.8) Male (%): 150 mg nilotinib (80); 300 mg nilotinib (71) H/Y stage: 3–5 Mean disease duration, y: 150 mg nilotinib (10.6); 300 mg nilotinib (12.4)	150 mg or 300 mg nilotinib once daily for 6 months	3 months	Tolerability, UPDRS III, α-synuclein, HVA, AEs
Pagan et al. (2020)	Single center, RCT, double blind, placebo controlled	75	Mean age, y: palcebo (68.6); 150 mg nilotinib (66.6); 300 mg nilotinib (70.0) Male (%): palcebo (84); 150 mg nilotinib (56); 300 mg nilotinib (80) H/Y stage: 2.5–3 Mean disease duration, y: palcebo (10.0); 150 mg nilotinib (12.3); 300 mg nilotinib (10.0)	150 mg or 300 mg nilotinib once daily for 12 months	3 months	Tolerability, MDS-UPDRS III, α-synuclein, HVA, DOPAC, AEs
Simuni et al. (2021)	Multicenter, RCT, double blind, placebo controlled	76	Mean age, y: palcebo (65.5); 150 mg nilotinib (61.2); 300 mg nilotinib (66.9) Male (%): palcebo (64); 150 mg nilotinib (60); 300 mg nilotinib (81) H/Y stage: 2–3 Mean disease duration, y: palcebo (9.4); 150 mg nilotinib (8.5); 300 mg nilotinib (11.7)	150 mg or 300 mg nilotinib once daily for 6 months	2 months	Tolerability, MDS-UPDRS III, HVA, DOPAC, AEs

### Risk of bias assessment

**Figure 2:**
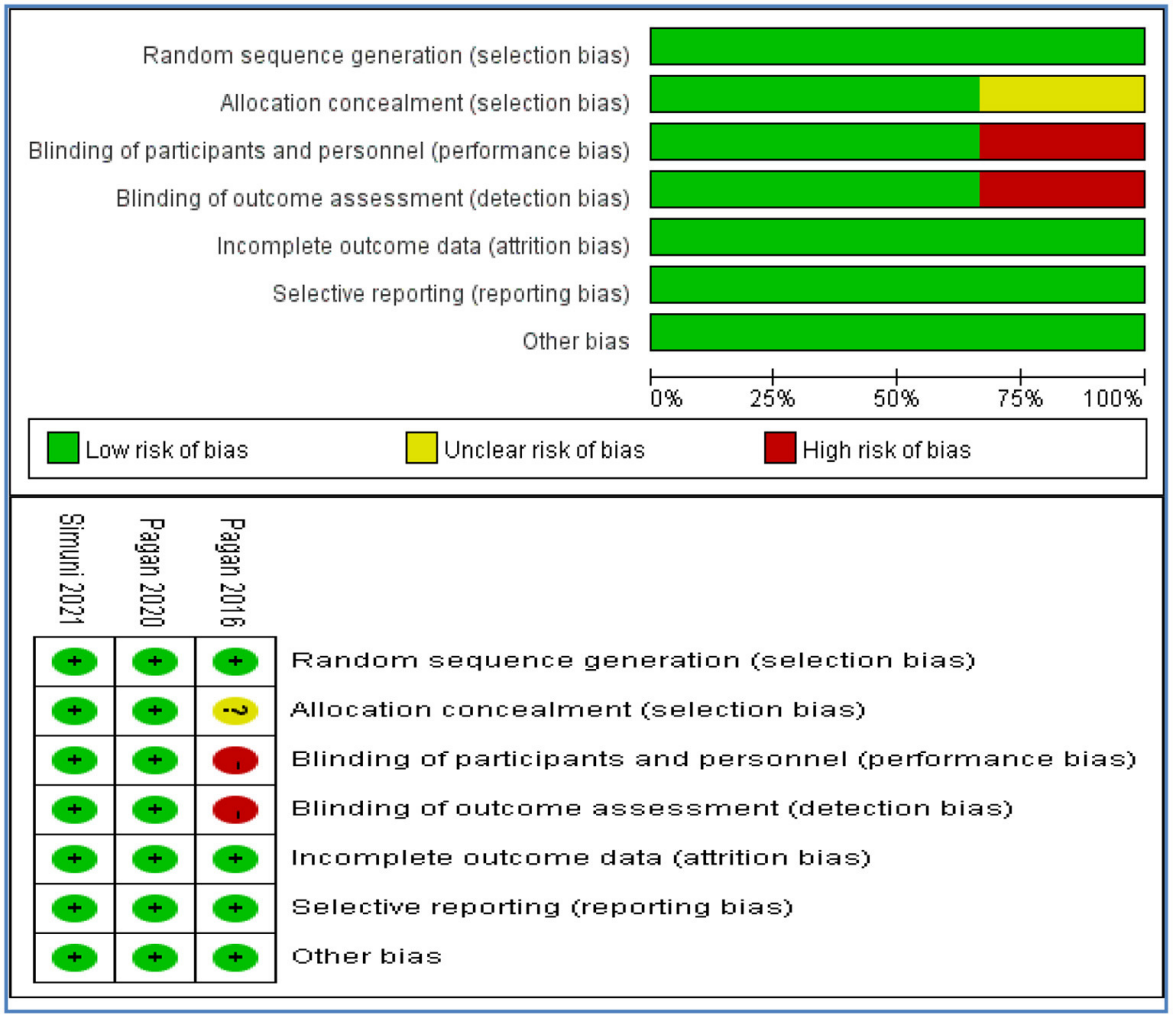
Risk of bias assessment.

Of the 3 included studies assessed for risk of bias, 2 were assessed at low risk on all assessed items. One study was assessed at high risk on performance and detection bias due to open label without blinding, and at unclear risk on selection bias without reporting the method of allocation concealment. The risk of bias was summarized in Figure 2.

### Outcomes

#### Tolerability

**Figure 3:**
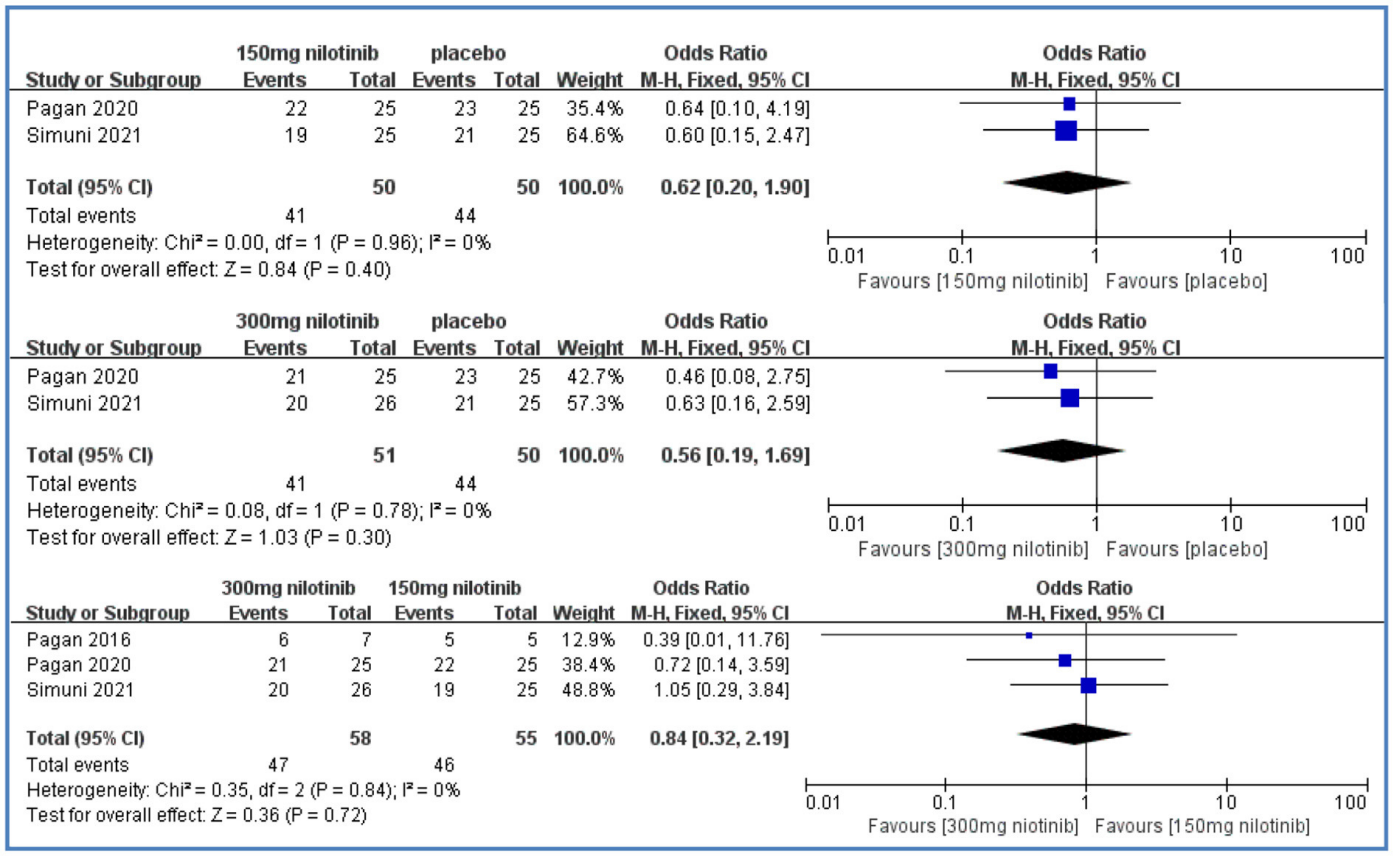
Forest plot of tolerability.

Tolerability was reported in three studies (Pagan et al., 2016, 2020; Simuni et al., 2021). Compared with placebo, there were no significant differences in the 150 mg nilotinib group [OR = 0.62, 95%CI = (0.20, 1.90), *P* > 0.05] and in the 300 mg nilotinib group [OR = 0.56, 95%CI = (0.19, 1.69), *P* > 0.05] and all low heterogeneity (I^2^ = 0%). There were also no significant differences between the 300 mg nilotinib group and 150 mg nilotinib group [OR = 0.84, 95%CI = (0.32, 2.19), *P* > 0.05] and low heterogeneity (*I*^2^ = 0%) (Figure 3).

#### Efficacy

##### MDS-UPDRS III

**Figure 4:**
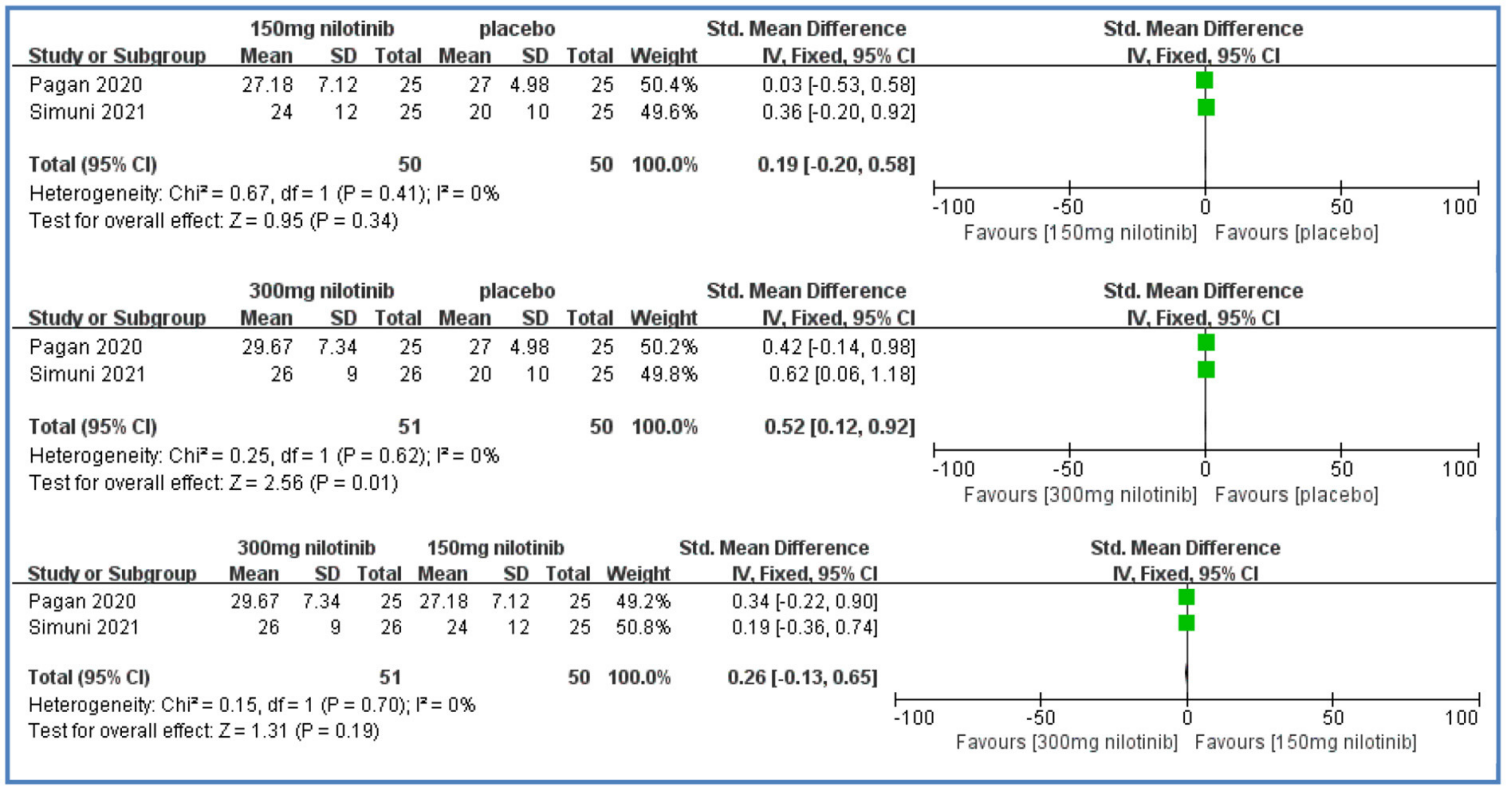
Forest plot of MDS-UPDRS III scores.

Two studies reported MDS-UPDRS III scores (Pagan et al., 2020; Simuni et al., 2021). Compared with placebo, there were significantly higher MDS-UPDRS III scores in the 300 mg nilotinib group [SMD = 0.52, 95%CI = (0.12, 0.92), *P* = 0.01] with low heterogeneity (*I*^2^ = 0%). There were no significant differences between the 150 mg nilotinib group and placebo [SMD = 0.19, 95%CI = (−0.20, 0.58), *P* > 0.05] with low heterogeneity (*I*^2^ = 0%); between the 300 mg nilotinib group and 150 mg nilotinib group [SMD = 0.26, 95%CI = (−0.13, 0.65), *P* > 0.05] with low heterogeneity (*I*^2^ = 0%) (Figure 4).

##### UPDRS III

Only 1 study reported UPDRS III scores in the 300 mg nilotinib group and 150 mg nilotinib group (Pagan et al., 2016). And no significant differences were found between the two groups [SMD = −0.45, 95%CI = (−1.62, 0.72), *P* > 0.05] (Supplementary Figure 1).

#### CSF biomarker levels

##### α-synuclein

Two studies reported α-synuclein levels (Pagan et al., 2016, 2020). We found lower α-synuclein levels in the 300 mg nilotinib group [SMD = −1.16, 95%CI = (−1.70, −0.61), *P* < 0.0001] when compared with the 150 mg nilotinib group, and low heterogeneity (*I*^2^ = 37%). And one study showed that the 300 mg nilotinib group had significantly lower α-synuclein levels than placebo [SMD = −2.16, 95%CI = (−3.38, −1.84), *P* < 0.00001] and that there were no significant differences between the 150 mg nilotinib group and placebo [SMD = 0.14, 95%CI = (−0.42, 0.69), *P* > 0.05] (Supplementary Figure 2).

##### HVA

All studies reported HVA levels. A pooled analysis showed no significant differences in the 150 mg nilotinib group [SMD = −0.08, 95%CI = (−1.26, 1.11), *P* > 0.05] with high heterogeneity (*I*^2^ = 88%) and in the 300 mg nilotinib group [SMD = −0.10, 95%CI = (−0.77, 0.57), *P* > 0.05] with high heterogeneity (*I*^2^ = 66%) when compared with placebo. There were also no significant differences between two nilotinib groups [SMD = −0.06, 95%CI = (−0.64, 0.51), *P* > 0.05] with high heterogeneity (*I*^2^=51%) (Supplementary Figure 3).

##### DOPAC

Two studies reported DOPAC levels (Pagan et al., 2020; Simuni et al., 2021). Compared with placebo, there were significantly higher DOPAC levels in the 300 mg nilotinib group [SMD = 0.52, 95%CI = (0.12, 0.92), *P* = 0.01] with low heterogeneity (*I*^2^ = 32%). There were no significant differences between the 150 mg nilotinib group and placebo [SMD = 0.18, 95%CI = (−0.92, 1.27), *P* > 0.05] with high heterogeneity (*I*^2^ = 87%) and between two doses of nilotinib [SMD = 0.39, 95%CI = (0, 0.78), *P* = 0.05] with low heterogeneity (*I*^2^ = 0%) (Supplementary Figure 4).

### Safety

Our pooled results showed no significant increase or decrease in the incidence of non-SAEs or SAEs of interest when comparing 150 mg nilotinib with 300 mg nilotinib and when comparing different doses of nilotinib with placebo (Supplementary Figures 5–10). Among non-SAEs, the most common were fall [17 of 50 (34%) in the 150 mg nilotinib group, 13 of 51 (25.5%) in the 300 mg nilotinib group], followed by musculoskeletal disorders [17 of 55 (30.9%) in the 150 mg nilotinib group, 10 of 58 (17.2%) in the 300 mg nilotinib group]. The incidences of skin and subcutaneous disorders, and gastrointestinal disorders were summarized in Supplementary Table 1. Among SAEs, the incidences of serious cardiac disorders and serious gastrointestinal disorders increased with higher dose of nilotinib, and were summarized in Supplementary Table 1.

## Discussion

This is the first meta-analysis comparing tolerability, efficacy, safety, and CSF biomarker levels at different doses of nilotinib in PD patients. Our study showed that neither 150 mg nor 300 mg nilotinib had beneficial clinical effects in the treatment of PD patients, except that 300 mg nilotinib could improve α-synuclein and DOPAC levels. Moreover, nilotinib had acceptable safety and tolerability with no significant differences in any comparison, which was consistent with the original studies. Our study showed that the incidence of serious cardiac disorders was correspondingly doubled when treated with double doses of nilotinib. Nilotinib has been warned with an increased risk of dose-related cardiac disorders, and the incidences are 9.9 and 15.9% among patients treated with nilotinib 300 mg twice daily, nilotinib 400 mg twice daily in the treatment of CML patients (Jabbour and Kantarjian, 2018). Therefore, this dose-related toxicity should be considered when conducting clinical trials with enlarged doses.

When treated with 150 mg nilotinib, it failed to provide an improvement in motor ability and CSF biomarker levels, possibly because the low concentration of nilotinib accumulated in the brain is not sufficient to inhibit c-Abl. Previous preclinical studies found that low doses of nilotinib had the ability to inhibit c-Abl, improve motor outcomes and CSF biomarker levels (Hebron et al., 2013a,b, 2014; Pagan et al., 2016). However, nilotinib does not appear these effects clinically because only a maximum of 10% of the concentration thought to be adequate to inhibit c-Abl was detected in the brain of PD patients (Pagan et al., 2019; Werner and Olanow, 2022). This may be interpreted by ATP-binding cassette (ABC) transporters which facilitate nilotinib removal from brain, therefore, nilotinib hardly achieves effective concentration to inhibit c-Abl. In addition, the duration of nilotinib to inhibit c-Abl in the brain is 6 hours, (Pagan et al., 2016) once-daily administration can not sustain the effect throughout the day.

When treated with 300 mg nilotinib, we found a conflict result that nilotinib could significantly worsen motor ability but significantly decrease α-synuclein levels and increase DOPAC levels, which was inconsistent with previous studies. These studies had demonstrated that nilotinib could accelerate autophagic clearance to degrade α-synuclein accumulated in the cells, protect dopaminergic neurons, increase dopamine and its metabolity DOPAC levels, and result in a motor improvement (Hebron et al., 2013a, 2014; Karuppagounder et al., 2014; Lonskaya et al., 2014; Wu et al., 2021). This confounding result between dose-dependent motor disability and improvement in CSF biomarker levels in our study may be due to the fact that all patients in three included studies were diagnosed with PD over 10 years with least H/Y stage 2 and all of them were treated with the concurrent chronic levodopa therapy. The ELLDOPA study, which aimed to assess the effect of levodopa on the progression of PD for a period of 42 weeks, found that levodopa significantly improved UPDRS scores in a dose-dependent manner, but this effect gradually diminished, eventually, UPDRS III worsened compared with baseline (Fahn et al., 2004). One of the potential mechanisms related to this variable effect may be the dopamine neurotoxicity caused by dopamine metabolites 2,4,5trihydroxyphenylalanine (TOPA) and TOPA-quinone, (LeWitt, 2015) which may counterbalance the neuroprotective effect of nilotinib. Nilotinib enters the brain through the blood-brain barrier in a dose-independent manner, and its inhibition of c-Abl is equivalent to that of 150 mg (Pagan et al., 2019). It is possible that the detrimental effects of chronic levodopa therapy on motor outcomes may conceal the minor clinical benefits of nlotinib on the inhibition of c-Abl, which may be an interpretation for this conflict.

In terms of HVA levels, we found nilotinib could nonsignificantly decrease HVA levels with high heterogeneity in any comparison. This result is consistent with the original study conducted by Simuni et al. (2021). However, in the open label study conducted by Pagan et al. (2016) CSF HVA levels significantly increased in the 150 mg nilotinib group at 2 months but not at 6 months, and it only significantly increased in the 300 mg nilotinib group at 6 months. In the double blind, placebo controlled study conducted by Pagan et al. (2020) CSF HVA levels significantly increased in the 150 mg nilotinib group at 12 months but not in the 300 mg nilotinib group. The variable HVA levels and differences between three original studies may be because of concurrent treatment with PD dopaminergic therapies, especially MAO-B inhibitors in the study conducted by Simuni et al., affecting dopamine metabolites and confounding results. High heterogeneity may result in different analytical methods and course of treatment. Study published by Pagan et al. (2016) used ELISA analysis. Pagan et al. (2020) and by Simuni et al. (2021) used LC-MS/MS analysis. Therefore, the results should be viewed with caution given the high heterogeneity.

Strengths of this systematic review and meta-analysis include that we performed a comprehensive literature search about this topic and this is the first meta-analysis based on all published RCTs. Apart from one study lack of blinding, other studies were of high quality. All outcomes had low heterogeneity except for HVA levels. And no publication bias was detected in our study. There are also several limitations in this meta-analysis. Firstly, only 3 relatively small RCTs with 163 patients were included. Secondly, one study included the patients with dementia with lewy bodies, which could impact the accuracy of results comparing different doses of nilotinib. Thirdly, some outcomes were reported in only 1 study, the pooled results should be carefully considered. Finally, the pooled results may be affected by data selected in different stages of treatment due to variable courses of treatment and length of follow-up. In our study, except for MDS-UPDRS scores selected in June, data of the last time node reported in the included studies were used for pooling.

Given the potential effects of chronic levodopa treatment on motor function and CSF biomarkers, further clinical trials should be conducted in patients with early PD who are not treated with levodopa to determine whether nilotinib stabilizes PD symptoms and/or its association with levodopa. Although nilotinib has shown well tolerability and safety, we still recommend low doses of nilotinib in further trials because of dose-related cardiac disorders. Pagan et al. (2020) found that 150 mg nilotinib significantly improved UPDRS III motor score at 15 months compared with baseline (−2.82 points), which was greater than that of the ELLDOPA study (1.4, 1.4, and −1.4 points for 150 mg, 300 mg, and 600 mg/ day, respectively) (Fahn et al., 2004). Due to the variable symptoms of PD, different PD management and care in multi-center studies can minimize its impact on results compared with single center studies. Pagan et al. (2020) and Simuni et al. (2021) both identified limitations of open-label studies on symptomatic results in PD. Taken together, further clinical trials should be conducted in strict accordance with protocols and criteria of randomized, double-blind, placebo-controlled, multicenter, large-sample trials over 15 months to investigate the effects of 150 mg or 300 mg nilotinib in early PD patients without levodopa use.

## Conclusion

Although our study demonstrated favorable tolerability and safety of different doses of nilotinib, and improvement in part of CSF biomarker levels of 300 mg nilotinib, the bad efficacy on motor outcomes indicated that nilotinib had no advantages in the clinic. These findings from three small sample-size trials should not be applied to a larger population. And stronger evidence from large-sample, well-designed trials in patients without chronic levodopa treatment is needed in the future.

## Data availability statement

The original contributions presented in the study are included in the article/Supplementary material, further inquiries can be directed to the corresponding authors.

## Author contributions

XX, PY, LK, XC, JL, and YL designed the study. XX, LK, and XC identified included studies, assessed risk of bias, extracted data, and performed data analysis. XX, PY, JL, and YL wrote and revised the manuscript. All authors contributed to the article and approved the submitted version.

## Funding

This work was supported by the Sichuan Provincial Department of Education (SCYG2020-04 and SCYG2019-04).

## Conflict of interest

The authors declare that the research was conducted in the absence of any commercial or financial relationships that could be construed as a potential conflict of interest.

## Publisher's note

All claims expressed in this article are solely those of the authors and do not necessarily represent those of their affiliated organizations, or those of the publisher, the editors and the reviewers. Any product that may be evaluated in this article, or claim that may be made by its manufacturer, is not guaranteed or endorsed by the publisher.
