# Supplementary material for: Nilotinib in Parkinson's disease: A systematic review and meta-analysis
Source: Front Aging Neurosci. 2022 Sep 29;14:996217. doi: 10.3389/fnagi.2022.996217 (PMC9558096; doi:10.3389/fnagi.2022.996217)
Supplement: Supplementary file 1 [file Data_Sheet_1.docx]

**Supplementary material**

Nilotinib in Parkinson’s disease: A systematic review and meta-analysis

**Search strategy for PubMed**

**Figure 1.** Forest plot of UPDRS Ⅲ scores

**Figure 2.** Forest plot of α-synuclein levels

**Figure 3.** Forest plot of HVA levels

**Figure 4.** Forest plot of DOPAC levels

**Figure 5.** Forest plot of fall

**Figure 6.** Forest plot of musculoskeletal disorders

**Figure 7.** Forest plot of skin and subcutaneous disorders

**Figure 8.** Forest plot of gastrointestinal disorders

**Figure 9.** Forest plot of serious cardiac disorders

**Figure 10.** Forest plot of serious gastrointestinal disorders

**Table 1.** The incidences of AEs

**Table 2.** Publication bias of tolerability

**Search strategy for PubMed**

("nilotinib"[Supplementary Concept] OR "nilotinib"[All Fields]) AND ("parkinson disease"[MeSH Terms] OR ("parkinson disease"[MeSH Terms] OR ("parkinson"[All Fields] AND "disease"[All Fields]) OR "parkinson disease"[All Fields]) OR ("parkinson disease"[MeSH Terms] OR ("parkinson"[All Fields] AND "disease"[All Fields]) OR "parkinson disease"[All Fields] OR ("idiopathic"[All Fields] AND "parkinson s"[All Fields] AND "disease"[All Fields]) OR "idiopathic parkinson s disease"[All Fields]) OR ("parkinson disease"[MeSH Terms] OR ("parkinson"[All Fields] AND "disease"[All Fields]) OR "parkinson disease"[All Fields] OR ("lewy"[All Fields] AND "body"[All Fields] AND "parkinson s"[All Fields] AND "disease"[All Fields]) OR "lewy body parkinson s disease"[All Fields]) OR ("parkinson disease"[MeSH Terms] OR ("parkinson"[All Fields] AND "disease"[All Fields]) OR "parkinson disease"[All Fields] OR ("parkinson s"[All Fields] AND "disease"[All Fields] AND "idiopathic"[All Fields]) OR "parkinson s disease idiopathic"[All Fields]) OR ("parkinson disease"[MeSH Terms] OR ("parkinson"[All Fields] AND "disease"[All Fields]) OR "parkinson disease"[All Fields] OR ("parkinson s"[All Fields] AND "disease"[All Fields] AND "lewy"[All Fields] AND "body"[All Fields]) OR "parkinson s disease lewy body"[All Fields]) OR ("parkinson disease"[MeSH Terms] OR ("parkinson"[All Fields] AND "disease"[All Fields]) OR "parkinson disease"[All Fields] OR ("parkinson"[All Fields] AND "disease"[All Fields] AND "idiopathic"[All Fields]) OR "parkinson disease idiopathic"[All Fields]) OR ("parkinson disease"[MeSH Terms] OR ("parkinson"[All Fields] AND "disease"[All Fields]) OR "parkinson disease"[All Fields] OR "parkinson s disease"[All Fields]) OR ("parkinson disease"[MeSH Terms] OR ("parkinson"[All Fields] AND "disease"[All Fields]) OR "parkinson disease"[All Fields] OR ("idiopathic"[All Fields] AND "parkinson"[All Fields] AND "disease"[All Fields]) OR "idiopathic parkinson disease"[All Fields]) OR ("parkinson disease"[MeSH Terms] OR ("parkinson"[All Fields] AND "disease"[All Fields]) OR "parkinson disease"[All Fields] OR ("lewy"[All Fields] AND "body"[All Fields] AND "parkinson"[All Fields] AND "disease"[All Fields]) OR "lewy body parkinson disease"[All Fields]) OR ("parkinson disease"[MeSH Terms] OR ("parkinson"[All Fields] AND "disease"[All Fields]) OR "parkinson disease"[All Fields] OR ("primary"[All Fields] AND "parkinsonism"[All Fields]) OR "primary parkinsonism"[All Fields]) OR ("parkinson disease"[MeSH Terms] OR ("parkinson"[All Fields] AND "disease"[All Fields]) OR "parkinson disease"[All Fields] OR ("parkinsonism"[All Fields] AND "primary"[All Fields]) OR "parkinsonism primary"[All Fields]) OR ("parkinson disease"[MeSH Terms] OR ("parkinson"[All Fields] AND "disease"[All Fields]) OR "parkinson disease"[All Fields] OR ("paralysis"[All Fields] AND "agitans"[All Fields]) OR "paralysis agitans"[All Fields]))

**Figure 1. Forest plot of UPDRS Ⅲ scores**

**
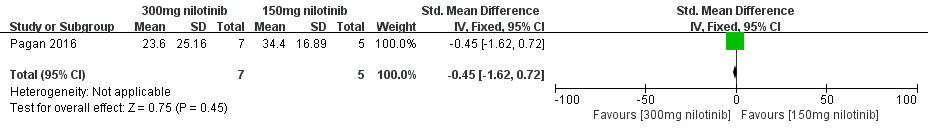
**

**Figure 2. Forest plot of α-synuclein levels**


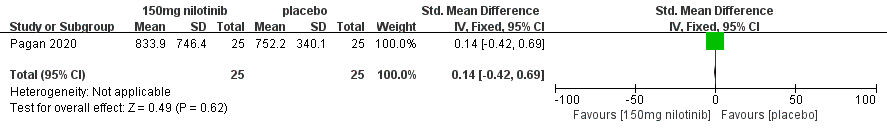

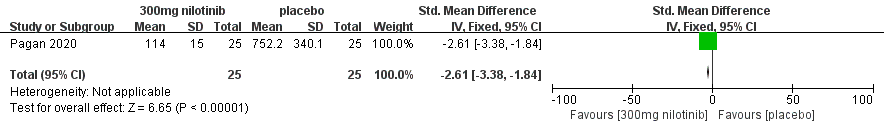

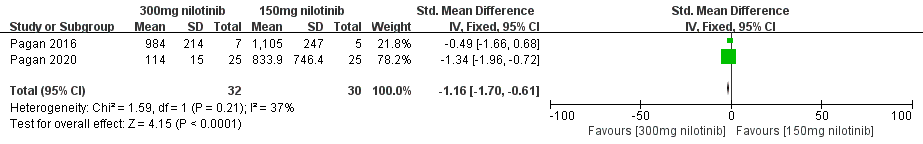


**Figure 3. Forest plot of HVA levels**


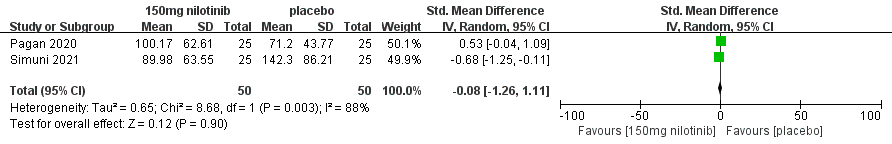

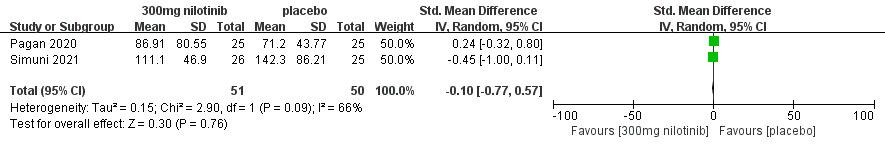

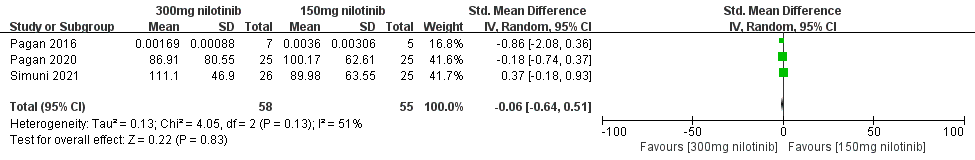


**Figure 4. Forest plot of DOPAC levels**


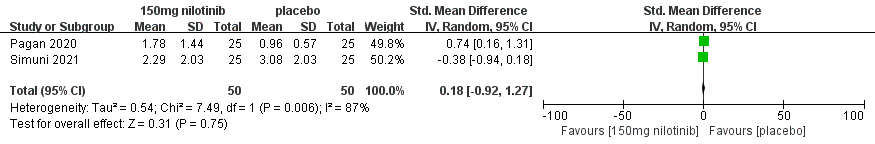

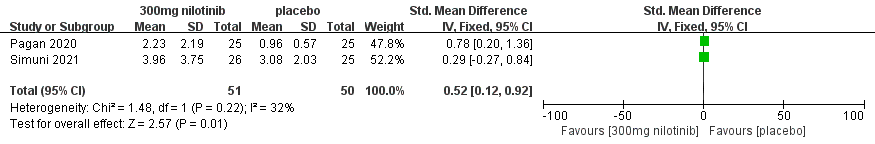

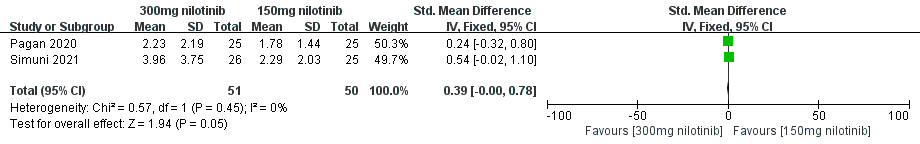


**Figure 5. Forest plot of fall**


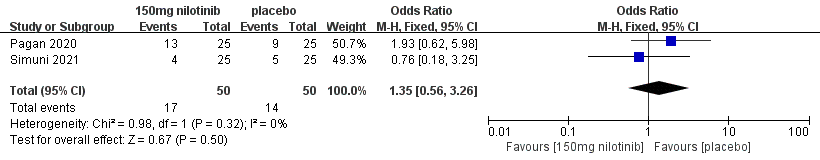

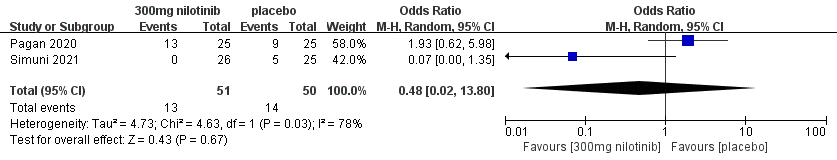

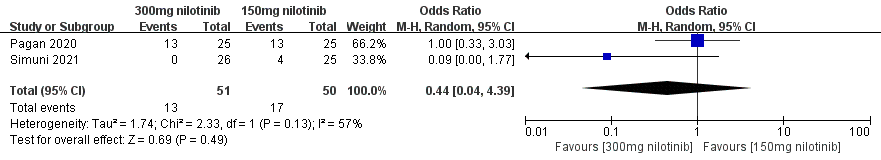


**Figure 6. Forest plot of musculoskeletal disorders**


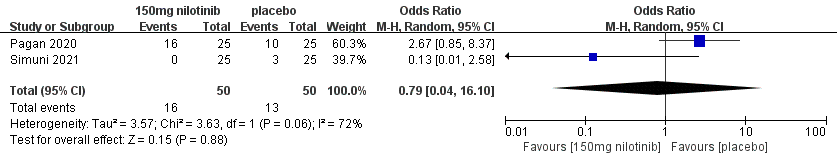

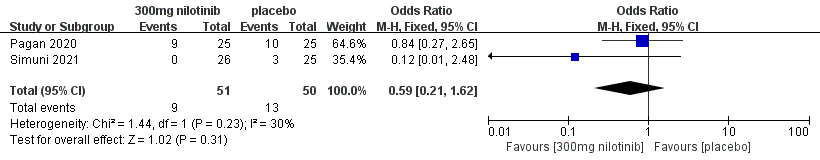

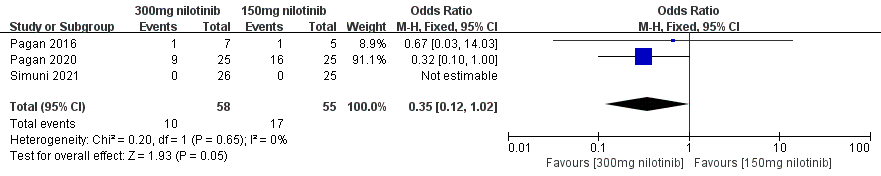


**Figure 7. Forest plot of skin and subcutaneous disorders**


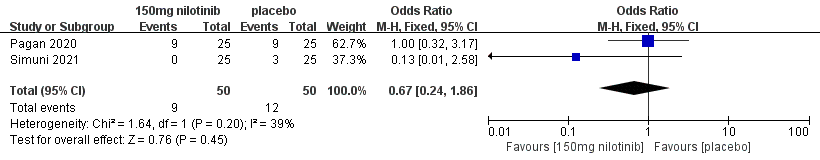

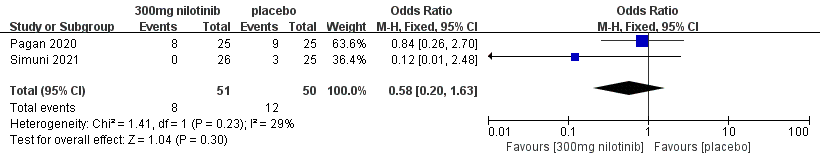

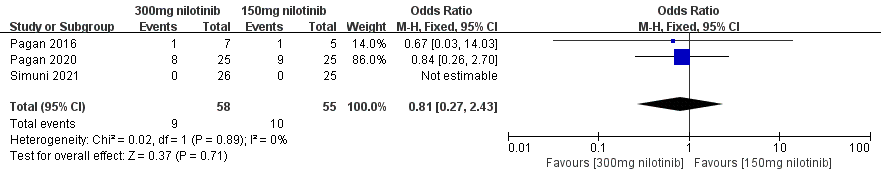


**Figure 8. Forest plot of gastrointestinal disorders**


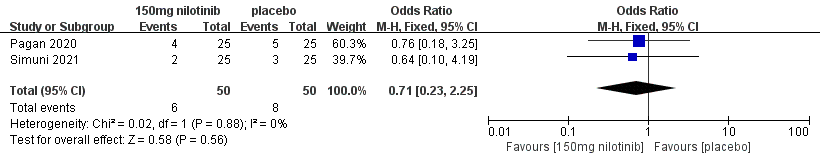

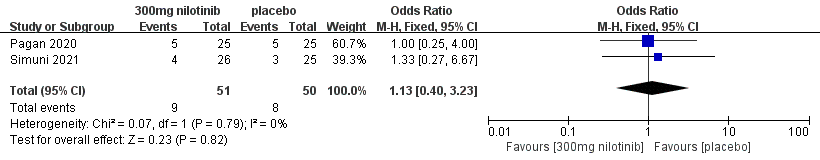

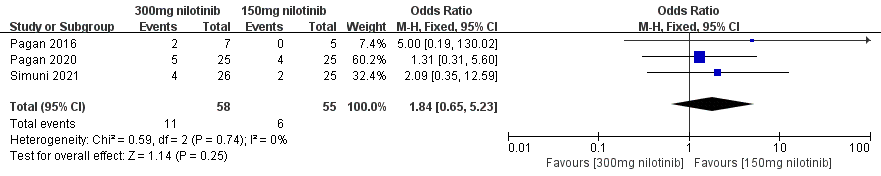


**Figure 9. Forest plot of serious cardiac disorders**


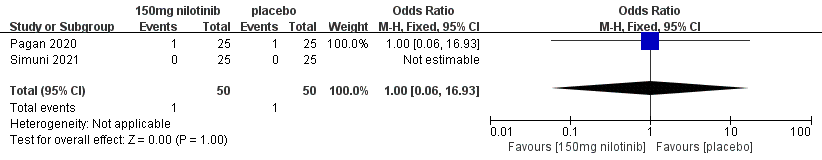

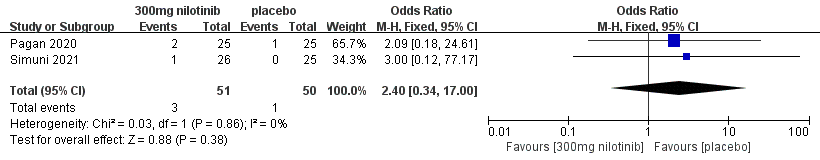

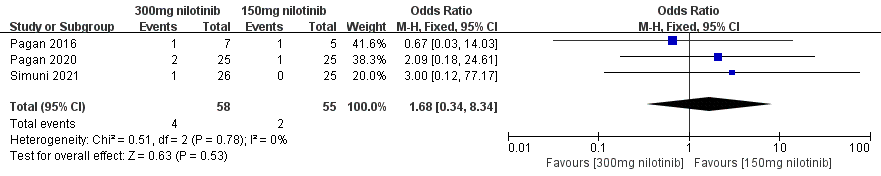


**Figure 10. Forest plot of serious gastrointestinal disorders**


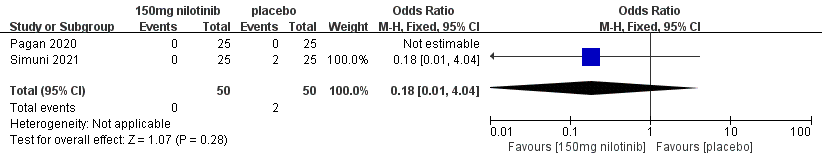

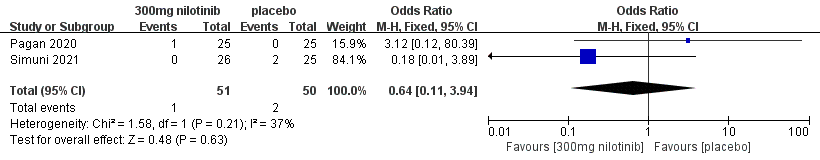

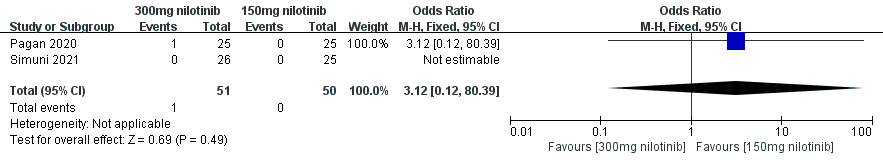


**Table. 1 The incidences of AEs**

| Outcomes | No. of studies | 150mg nilotinib | 300mg nilotinib |
| --- | --- | --- | --- |
| Non-SAEs |  |  |  |
| Fall | 2 | 34.0% (17/50) | 25.5% (13/51) |
| Musculoskeletal disorders | 3 | 30.9% (17/55) | 17.2% (10/58) |
| Skin and subcutaneous disorders | 3 | 18.2% (10/55) | 15.5% (9/58) |
| Gastrointestinal disorders | 3 | 10.9% (6/55) | 19.0% (11/58) |
| SAEs |  |  |  |
| Serious cardiac disorders | 3 | 3.6% (2/55) | 7.0% (4/58) |
| Serious gastrointestinal disorders | 2 | 0.0% (0/50) | 2.0% (1/51) |

**Table. 2 Publication bias of tolerability**

|  | Egger test | Begg rank correlation |
| --- | --- | --- |
| P value | 0.296 | 0.358 |
